# An empirical exploration of the diversified R ecosystem

**DOI:** 10.1371/journal.pone.0346017

**Published:** 2026-04-06

**Authors:** Tian-Yuan Huang, Zhilan Lou

**Affiliations:** School of Data Science, Zhejiang University of Finance and Economics, Hangzhou, China; Max Planck Institute for Solid State Research, GERMANY

## Abstract

Born in the late 20th century, R has become one of the most widely used software environments for statistical computing and graphics, undergoing substantial transformation alongside advances in information technology and the rise of data-intensive research. By integrating large-scale usage data from the Comprehensive R Archive Network (CRAN) with bibliometric records from the Scopus database, this study provides a comprehensive empirical review of the R ecosystem between 2005 and 2024. We examine long-term trends in R adoption, the functional structure and popularity patterns of its package ecosystem, disciplinary applications in academia, and collaboration behaviors within the developer community. The results reveal sustained growth in both R software and package downloads, with package usage showing more stable and continuous expansion over time. A keyword co-occurrence analysis indicates that the ecosystem is organized around a dense statistical core, closely connected with diverse modeling frameworks, modern machine learning techniques, and application-oriented functionalities. Bibliometric evidence further demonstrates the widespread and growing adoption of R across scientific disciplines, particularly in agricultural and biological sciences, environmental science, medicine, and the social sciences. In addition, collaboration analysis shows that multi-author packages are more prevalent and tend to achieve greater reuse and higher download activity, highlighting the role of collective development in sustaining the vitality and long-term relevance of the R ecosystem. Overall, these findings position R as a resilient, community-driven platform whose evolution continues to be shaped by interdisciplinary collaboration and open-source innovation.

## Introduction

Among the various programming languages, R [1] is famous for its capability in data mining. In recent decades, R’s position in TIOBE has shown notable fluctuations, including periods where it fell out of the top ranks before rising again (https://www.tiobe.com/tiobe-index/r/). Such drops have raised concerns among some observers that R’s relative popularity may be challenged by broader-purpose languages like Python, especially as industry demand and search interest shift toward languages with wider adoption in production systems. Even though R remains strong in statistics and research contexts, the variability in its ranking highlights the need to pay attention to how trends in tooling and community interest evolve over time rather than assuming static dominance.

As a programming language, R’s free open-source environment was derived from S language initiated at Bell Labs. Containing some people associated with S and S-PLUS, the R Core Team was founded in 1997 to develop and maintain R source code. The public management of R packages is mainly supported by CRAN, other major repositories include BioConductor, R-Forge and GitHub [2,3]. While these repositories have overlapped packages, CRAN remains the center of R package management system with least dependencies [4]. The usage of R is extensive, and gradually it becomes one of the most popular software in scientific research. This leads scientists to pay attentions to the scientific usage of R packages. Related researches include the focus on co-mention network of R packages [5] and citation patterns on R packages [6,7]. In addition, numerous studies have showed that researchers and practitioners from various backgrounds are embracing R for its openness and reproducibility [8–13].

In this paper, we would like to make an empirical review of R ecosystem based on the ever-improving archived document from CRAN (https://cran.r-project.org/) and the bibliometric database provided by Scopus (https://www.scopus.com/). We try to explore different components (including developers and users) in the R community, and discuss how they might interact with each other. Specifically, we implement our investigation from four aspects: (1) How overall user activity and adoption of R are changing over time? What functionalities is R providing us? (2) What are the most popular packages in R in the following 20 years since 2005? (3) Who are the major users of R in academia? What are the potential trends of R development in scientific community? (4) What is the general collaboration pattern of R community? By exploring these questions and making critical discussions, we intend to gain better understanding of the current R ecosystem and shape the future development of R.

### Role of R in the programming landscape

R is a powerful programming language primarily designed for statistical computing, data analysis, and visualization. To understand R’s role in the programming landscape, it’s helpful to categorize languages based on their primary use cases [14]. General-purpose languages like Python, Java, and C++ are known for their versatility, enabling a wide range of applications from web development to system programming. In contrast, domain-specific languages such as R, MATLAB, and SAS are tailored for specific fields. R is positioned as a leading tool in the realm of data analysis, where it competes most directly with Python [15]. Python, with its vast libraries like Pandas, NumPy, and Scikit-learn, is often chosen for its simplicity and the ability to integrate with other programming tasks, making it a strong competitor. However, R’s extensive collection of specialized packages, such as ‘dplyr’ for data manipulation and ‘ggplot2’ for visualization, often provides a significant advantage in data cleaning and graphical output. Other competitors include SAS, a commercial software suite widely used in industry for statistical analysis, and MATLAB, which is favored in academia and engineering for numerical computing but lacks R’s depth in statistical applications.

R’s strengths are rooted in its specialization. It boasts a comprehensive ecosystem of packages that cover almost every aspect of statistical analysis and data visualization. The language’s syntax, while initially challenging, is tailored to data operations, making complex analyses more intuitive for statisticians and data scientists [16]. R’s ability to produce high-quality graphics and visualizations is unmatched, which is crucial for interpreting data insights. Additionally, R has a strong, active community, ensuring continuous development, extensive documentation, and support. However, R is not without its weaknesses. One of the most significant drawbacks is its performance. R can be slower and less efficient with memory, particularly when handling large datasets, compared to more optimized languages like Python or C++. Additionally, compared with statistical software not requiring programming through a command-line interface, base R’s syntax can be difficult for beginners, especially those without a strong background in statistics. The language is less versatile for general-purpose programming, making it less ideal for projects that extend beyond data analysis.

In summary, R is a highly specialized tool that excels in statistical analysis and data visualization, making it the preferred choice for data scientists and statisticians. However, its limitations in speed, memory efficiency, and general-purpose programming mean that it often shares the spotlight with Python, especially in data science projects that require integration with other programming tasks.

## Methods

To get a comprehensive view on R ecosystem, multiple data sources are collected and utilized in our study. These datasets include: (1) Download information of R and R packages: Download logs of R and R packages could be accessed from RStudio CRAN Mirror (http://cran-logs.rstudio.com/), and information of daily download times could be retrieved via APIs from ‘cranlogs’ package [17]. While the RStudio CRAN Mirror is not the only option, it effectively reflects the overall situation due to its widespread popularity among RStudio users; (2) Meta data of R packages on CRAN: The meta data of R packages, such as their authors, maintainers, published date and imported packages, are archived in CRAN. These data could be extracted using ‘RWsearch’ package [18]. The target data in our research was retrieved in December, 2025; (3) Bibliometric data of papers citing R: The bibliometric data could help us explore how R is utilized in academia. Referring to the previous research [6], our study retrieved the bibliometric data of papers citing R software from Scopus database using advanced search (the advanced query was “REF ({R: A Language and Environment for Statistical Computing} OR {http://www.r-project.org}) AND DOCTYPE (ar)”), with the searches conducted separately for each subject area from 2005 to 2024. The ‘rscopus’ package [19] was used to facilitate the acquisition of bibliometric data from Scopus database. The bibliometric data contains information of publication year, title, keywords, journal title and ISSN, etc. Scopus Subject Area categories were used to classify the publications into different subjects.

Based on these data sources, we further focused on the semantic content of R packages to explore the initiatives underlying R package development. Specifically, we extracted the keywords from the “description” field of R packages listed on CRAN and constructed a keyword co-occurrence network to explore the initiatives of developing R packages. To complete this task, we have: (1) Used n-gram tokenizer to segment the corpus. The maximum and minimum of n were 5 and 2 respectively. Unigrams were excluded because they are too granular to carry accurate information. This process yielded the n-grams for each R package. (2) Filtering the n-grams using a user-defined dictionary based on literature keywords. We have used a reverse query to get all the literature that citing R software in Scopus database, then used the author keywords of these literature to form a R-related dictionary. Then the n-grams yielded in the previous step would be filtered by the dictionary (only phrases in the dictionary were retained). (3) Merge synonyms within the R package. Keywords with same stem would be merged into its most frequent form. In addition, if a keyword is a subset of another, they would be merged into the shorter one. For instance, “time series” and “time series analysis would be merged into “time series” (but they would not be merged into “time” because we have excluded unigrams). (4) Construction and visualization of knowledge graph based on keyword co-occurrence in R packages. Nodes in the network is the keywords of the R packages, edge between nodes means the two keywords have co-occurred in the description of the same package. Both the frequency and degree of the keywords were summarised and displayed in the visualization. The data analyses were carried out in R software (version 4.5.1). The R packages used in the study include ‘tidyverse’ [20], ‘data.table’ [21], ‘patchwork’ [22], ‘graphlayouts’ [23], ‘lubridate’ [24], ‘fst’ [25], ‘dtplyr’ [26], ‘akc’ [27] and ‘tidyfst’ [28].

## Results

### Temporal dynamics and functional scope of R

**Fig 1:**
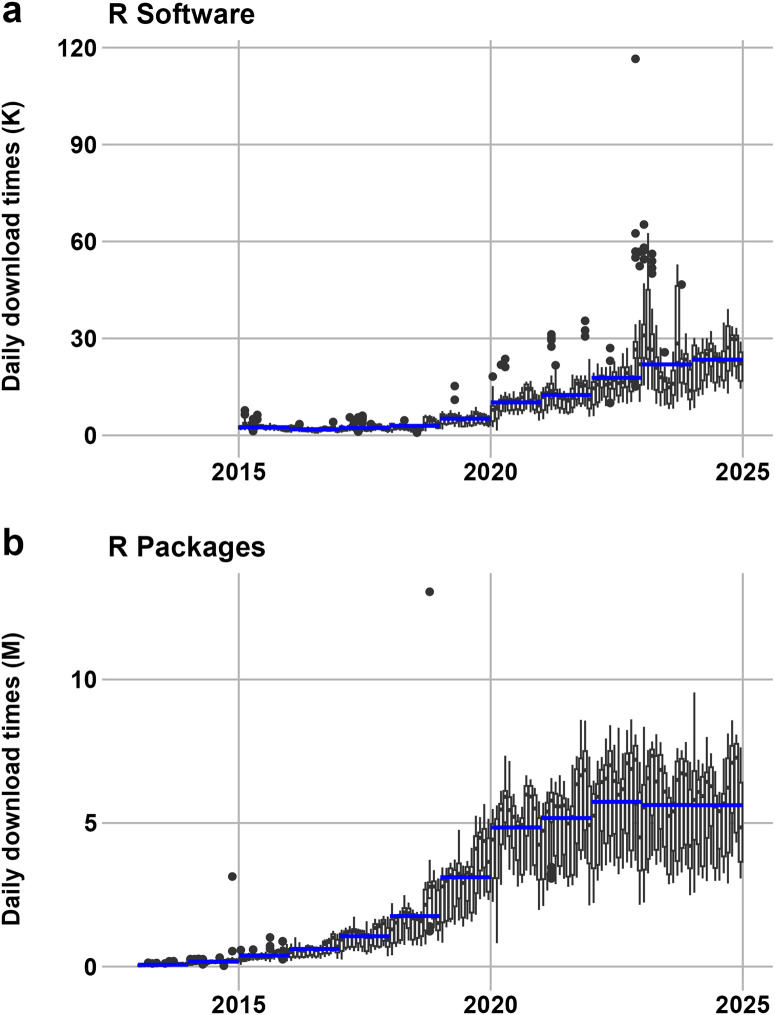
The daily downloads of R software (a) and R packages (b). The boxplots show the daily download times within the month. The blue lines show the average daily download in each year. Source of data: http://cran-logs.rstudio.com/.

Our analysis illustrates the temporal evolution of daily downloads of R software (Fig 1a) and R packages (Fig 1b). Overall, both series exhibit a clear long-term upward trend, indicating sustained growth in user activity and adoption of the R ecosystem. For R software, daily downloads remained at a relatively low and stable level between 2015 and 2018, followed by a pronounced increase after 2019. The annual average lines show stepwise growth, with several extreme peaks appearing in recent years, suggesting that major releases or external events can trigger short-term surges in downloads. In contrast, the growth pattern of R packages is more continuous and smoother. Package downloads increase almost monotonically over time, with particularly rapid expansion around 2019–2020, after which they stabilize at a higher level with substantial variability. The monthly boxplots reveal that the dispersion of daily downloads widens over time for both R software and packages, especially in the later years, reflecting an expanding and increasingly heterogeneous user base. Taken together, these patterns indicate that while overall adoption of R has accelerated markedly in the past decade, the software itself shows episodic growth, whereas R packages demonstrate a more stable and sustained expansion of the ecosystem’s functional use.

**Fig 2:**
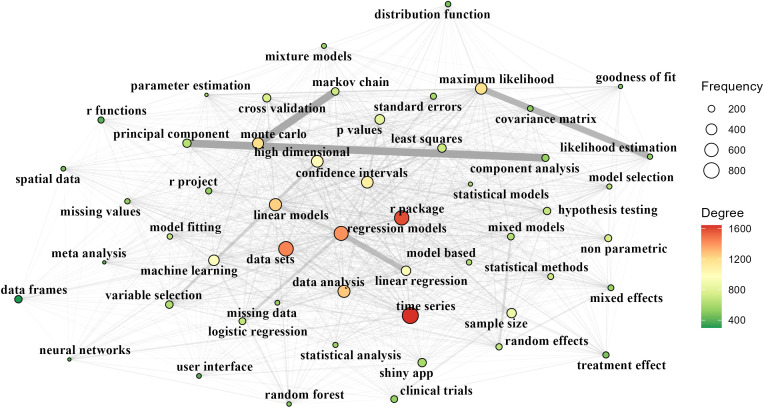
Knowledge graph of R based on package description archived on CRAN. Only top 50 keywords with largest frequency are displayed. The width of edge is proportional to the co-occurrence number.

Fig 2 shows the keyword co-occurrence network extracted from R package descriptions, in which node size and color represent degree and edges indicate co-occurrence relationships. The network is structured around a dense statistical and data-analytic core, with highly connected keywords such as “time series”, “data sets”, “regression models”, “data analysis”, and “linear models” occupying central positions, indicating their frequent co-occurrence with a wide range of other topics. This is not surprising, as R was first created by statisticians and designed to be a freely available language and environment for statistical computing and graphics [29]. Closely linked to this core are inferential and computational concepts including “maximum likelihood”, “monte carlo”, “confidence intervals”, “p values”, and “sample size”, highlighting the central role of statistical inference and estimation. A variety of modeling frameworks (such as “linear regression”, “logistic regression”, “mixed models”, “random effects”, and “non parametric”) form overlapping clusters, suggesting that different approaches are often combined within packages. Modern analytical techniques, represented by keywords like “machine learning”, “random forest”, and “high dimensional”, are embedded within the same network rather than appearing as isolated components, while application-oriented and implementation-oriented terms such as “shiny app”, “clinical trials”, and “missing data” appear as smaller but well-connected nodes. Overall, our result depicts an R package ecosystem organized around a strong statistical foundation, with diverse methods, applications, and user-oriented functionalities tightly interconnected through shared data-analytic themes.

### Popular R packages

**Fig 3:**
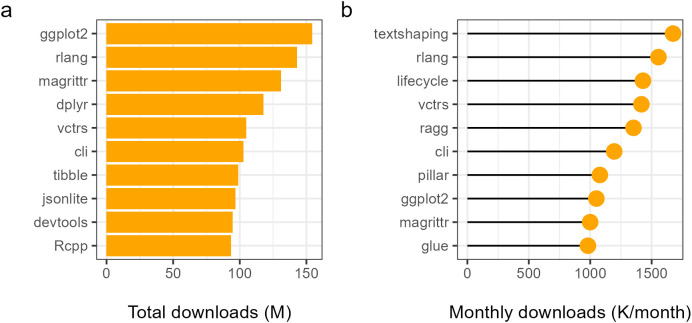
Top 10 R packages from 2005 to 2024. Based on: **(a)** Total downloads during the period. **(b)** Monthly downloads by average during the period.

In this section, we present the top 10 R packages ranked by total downloads (Fig 3a) and by average monthly downloads (Fig 3b), which capture two different dimensions of popularity. Total downloads reflect long-term cumulative adoption over the entire study period, whereas average monthly downloads indicate the intensity of usage during a package’s active lifespan. For Fig. 3b, the average is calculated starting from the release date of each package, and months with zero downloads prior to release or after archiving are excluded. This ensures that the metric reflects actual usage intensity rather than underrating newer packages.

The results show that a small group of core packages consistently dominates download activity, highlighting their central role in the R ecosystem. Packages such as ‘ggplot2’, ‘rlang’, ‘magrittr’, and ‘dplyr’ appear prominently, reflecting their widespread adoption across both end users and package developers. Their high cumulative download counts indicate sustained relevance over time, while strong monthly download levels suggest continued and active use in recent years. A closer inspection reveals differences in emphasis between cumulative and current usage. Packages like ‘ggplot2’ and ‘dplyr’ rank highly in total downloads, underscoring their long-standing importance in data visualization and data manipulation. These packages provide user-friendly, consistent APIs that lower the barrier to complex analytical tasks, making them indispensable tools for everyday data analysis. In contrast, the monthly download rankings place greater weight on infrastructure and developer-oriented packages, including ‘rlang’, ‘vctrs’, ‘lifecycle’, ‘pillar’, and ‘cli’. These packages are often used as dependencies rather than directly by end users, yet their high monthly download volumes indicate intense and ongoing reuse within the package development ecosystem.

Notably, packages such as ‘textshaping’ and ‘glue’ appear among the top performers in monthly downloads, reflecting the growing importance of text processing, formatting, and low-level utilities in modern R workflows. Their prominence suggests that R is increasingly used not only for statistical analysis, but also for producing polished outputs and building robust software tools. Similarly, the strong presence of packages like ‘vctrs’ and ‘rlang’ highlights the consolidation of modern programming paradigms in R, including tidy evaluation and consistent data structures, which have become foundational for many contemporary packages.

Overall, it could be found that the popularity of R packages is shaped by both user-facing functionality and behind-the-scenes infrastructure. While well-known analytical packages accumulate large total download counts over time, developer-focused packages dominate monthly usage due to their role as core dependencies. Together, these patterns demonstrate the maturity and complexity of the R ecosystem, in which a relatively small set of foundational packages supports a wide range of analytical, programming, and software development activities.

### Application of R in academia

**Fig 4:**
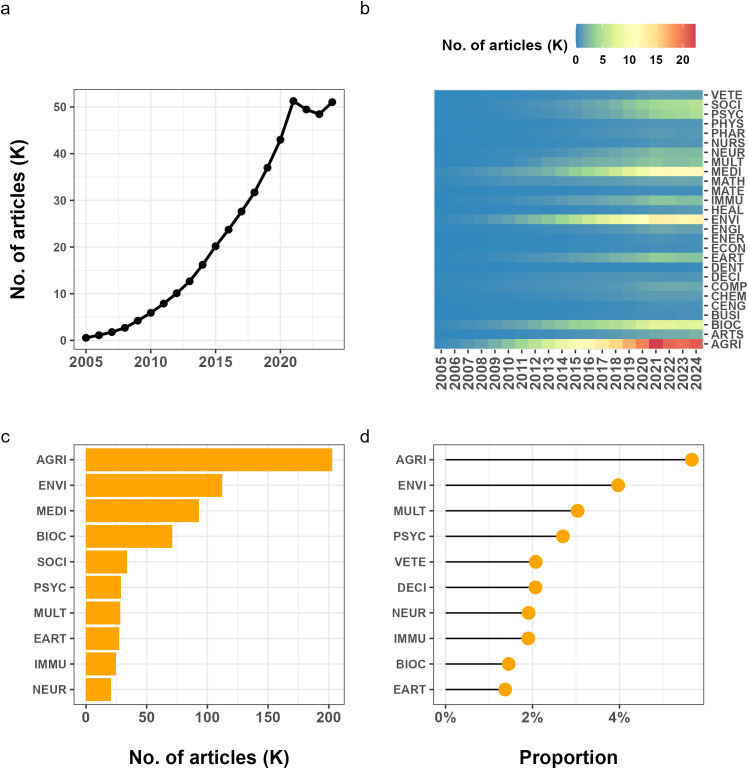
Usage of R in academia between 2005 and 2024. (a) Growth of academic articles citing R software. (b) The number of articles citing R software in different subject areas over time. (c) Top 10 subjects with most articles citing R software. (d) Top 10 subjects with most proportion of articles citing R software (calculated as the number of R-citing articles divided by the total number of publications in that subject area in Scopus). The subject classification is based on the list of Scopus Subject Area (AGRI: Agricultural and Biological Sciences; ARTS: Arts and Humanities; BIOC: Biochemistry, Genetics, and Molecular Biology; BUSI: Business, Management, and accounting; CENG: Chemical Engineering; CHEM: Chemistry; COMP: Computer Science; DECI: Decision Sciences; DENT: Dentistry; EART: Earth and Planetary Sciences; ECON: Economics, Econometrics, and Finance; ENER: Energy; ENGI: Engineering; ENVI: Environmental Science; HEAL: Health Professionals; IMMU: Immunology and Microbiology; MATE: Materials Science; MATH: Mathematics; MEDI: Medicine; MULT: Multidisciplinary; NEUR: Neuroscience; NURS: Nursing; PHAR: Pharmacology, Toxicology, and Pharmaceutics; PHYS: Physics and Astronomy; PSYC: Psychology; SOCI: Social Sciences; VETE: Veterinary).

According to the Scopus database, the number of academic articles citing R software was increasing from 2005 to 2024 (Fig 4a). While only 558 were tracked in 2005, the number of articles citing R software has reached 51,034 in 2024. Starting from a very low baseline in 2005, the annual number of articles grew steadily throughout the following years, with particularly rapid growth after 2015, and reached a high and relatively stable level in the early 2020s, indicating the widespread and mature adoption of R in academic research. A slight decline in 2021 may possibly reflect a normalization following the exceptional surge observed in 2020, when the early stage of the COVID-19 pandemic triggered an unusual expansion of data-intensive research activities. In this sense, the 2020 peak could represent a short-term spike, with 2021 marking a return to a more sustainable growth trajectory rather than a structural decline in the adoption of R.

When examining the disciplinary distribution of these articles, we could find clear differences across subject areas as well as consistent upward trends over time. R usage expanded across a broad range of disciplines, with especially strong growth observed in “Agricultural and Biological Sciences”, “Biochemistry, Genetics, and Molecular Biology”, “Earth and Planetary Sciences”, “Environmental Science”, “Immunology and Microbiology”, “Medicine”, “Psychology” and “Social Sciences” (Fig 4b).

To further distinguish between disciplinary scale and relative adoption, Fig 4c reports the absolute number of articles citing R in each subject area, whereas Fig 4d presents the proportion of R-citing articles relative to the total number of publications within the same subject area in Scopus (2005–2024). During the 20-year period, “Agricultural and Biological Sciences” and “Environmental Science” emerged as the most active subject areas using R. In absolute terms (Fig 4c), they published the largest numbers of R-related articles (202,881 and 112,204 respectively). In relative terms (Fig 4d), they also showed the highest proportions of R usage, accounting for 5.7% and 4.0% of all publications within their respective subject areas. By contrast, although “Computer Science” and “Mathematics” are conceptually close to R, they exhibit both lower absolute counts (14,460 and 16,767) and lower proportions (0.5% and 0.7%, respectively) during 2005–2024. This suggests that while R is widely adopted in empirical and data-intensive disciplines, its relative share is smaller in fields where multiple software ecosystems coexist. It is worth noting that statistics, which is closely related to R, is not listed as a standalone primary subject area in Scopus. Instead, it appears as subcategories within “Mathematics” (“Statistics and Probability”) and “Decision Sciences” (“Statistics, Probability and Uncertainty”), reflecting its interdisciplinary positioning.

### Collaboration patterns in R community

**Fig 5:**
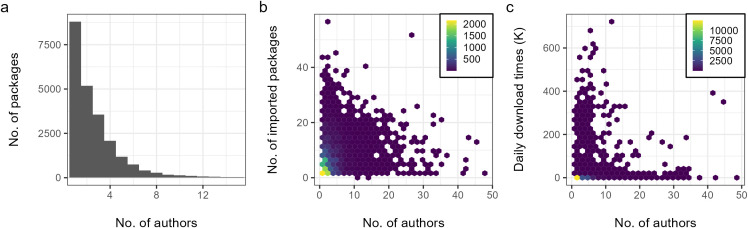
Collaboration patterns in R community. **(a)** Distribution of R package author number (packages with more than 15 authors are omitted in the visualization). **(b)** Correlation between author number and number of imported packages. **(c)** Correlation between author number and daily download times in 2024.

Examining the number of co-authors per package sourced from CRAN, it can be found that the package author number follows a long-tail distribution (Fig 5a). By the year of 2024, 8798 packages are single-authored, followed by 5179 with two, 3558 with three, 2076 with four and 1187 with five. For the rest, there are 2231 packages written by more than five authors. On average, each package is authored by 2.84 authors, with a median of 2. Generally, we could find more multi-authored packages than single-authored packages (14231 v. 8798). In examining the potential effects of collaboration (Fig 5b and 5c), we discovered a positive correlation between the number of package authors and the number of imported packages (Pearson, r = 0.20, p < 0.001***), and daily download times (Pearson, r = 0.12, p < 0.001***). This indicates that the collaboration behavior of R developers might possibly help to reuse and integrate more sources in R community. Moreover, the R package coming from team work might gain more popularity as more members are involved. This phenomenon is also common in the academia, where collaborative study attracts more citations than comparable solo research [30].

## Discussion

**Fig 6:**
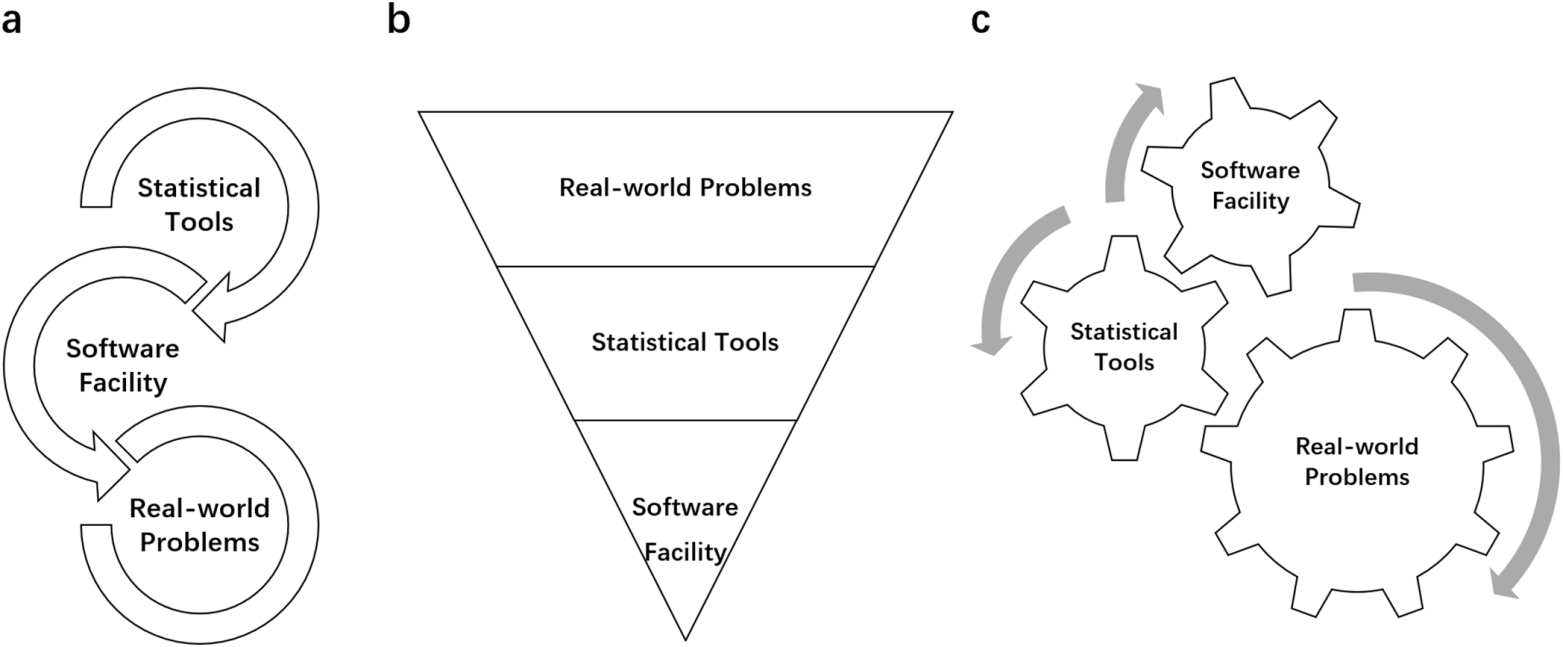
The ecosystem of R from different perspectives. **(a)** From perspective of statistics. **(b)** From perspective of computer science. **(c)** From perspective of practitioners.

In history, R was first designed by Ross Ihaka and Robert Gentleman, both statisticians interested in computer programming, as a personal project to build statistical tools in the teaching laboratory [31]. It could be regarded as the fruit of statistics and computer science. Therefore, the development of R has a deep root in statistics, this could also be found in today’s R ecosystem (Fig 2). Usually, statistical researchers would propose new ideas and implement these ideas in R, and then tested them in the real world (Fig 6a). A good package designed by statisticians provides the users with easy-to-use APIs and try to guide them to discover more with the additional settings provided by parameters. In this way, even users without any background knowledge could utilize the cutting-edge statistical tools in their work. At the same time, they might discover more issues at the usage and give feedbacks to the package maintainers, which help improve the statistical research in return. It is good practice for developers to keep good documentations for their work.

While R is initiated by statistics, the recent years have seen lots of popular packages designed for not-so-statistical tasks, such as high-performance computing, string operations and connection to other software. For example, Posit, the company behind RStudio, is actively working to unify R and Python within a single environment, addressing the growing demand for cross-language support in data science. Moreover, recent updates to R packages have enhanced the integration of ‘tidyverse’ tools with SQL, using packages like ‘dbplyr’, ‘odbc’, and ‘DBI’. This allows users to apply ‘tidyverse’ functions directly to SQL-based databases, streamlining the workflow between R and SQL. Beyond these computational tools, R has also expanded its utility by enabling the creation of interactive web applications through Shiny (https://shiny.posit.co/) and dynamic, reproducible reports with RMarkdown (https://rmarkdown.rstudio.com/) and Quarto (https://quarto.org/). These tools allow users to build interactive data-driven applications and multi-language documents, enhancing data engagement, collaboration, and the sharing of analyses across various formats. Generally, these tools could be categorized as computer facilities, and it is likely that these facilities have acted as footstones in the physical sense (Fig 6b). According to our investigation, the usage of R in computer science is relatively infrequent in academia. As R gets more popular in multiple fields, developers from different backgrounds are considering building a more comprehensive ecosystem for it. The early developers of R might never imagine that one day this light-weighted software could carry out complicated computations on million rows of data within minutes, and at the same time storing and exporting these results to dashboards in expressive graphics and tables. In the field of data science, the versatile R is not in the least inferior to Python, which is well-known for its “glue” feature. On the other hand, while there are lots of statistical tools wrapped in R packages, end-users might find it difficult to use them directly in the real world. The computer facilities, serving as a bridge between statistical tools and real-world problems, could fill this gap. For instance, some advanced statistical methods might be so far only accessible in R, but the real data are stored in different file formats (doc, pdf, tiff, json, etc.). It is worth noting that R has long been equipped with a robust set of packages that allow users to read and work with a variety of data formats, such as ‘pdftools’ for PDF, ‘jsonlite’ for JSON, and many others. These tools are so popular that they might gain more attention than other specific statistical tools, because the functionalities they provide are considered to be more general and irreplaceable in the workflow, whereas R provides various alternatives in statistical methods. In the future, the R community should attract more talents with a significant computer science background. The sparks between computer science and statistics would raise more amazing revolutions in the community and lift the ecosystem of R to a higher level.

Although R is rooted in statistics and developed by computer techniques, as the concept and practice of big data swept the world, the fruit of R is shared on a much broader scale. We could find that in academia, a huge group of researchers from various fields are benefiting from R. Usually, they start their research from real-world problems, making hypothesis and seek for the right tools to provide evidence (Fig 6c). One major reason is, these scientific areas are all embracing the advent of big data and moving toward evidence-based qualitative science, and R turns out to be one of the most appropriate tools for this trend. While science emphasizes the academic results should be reproducible, the documented R scripts could provide evidence for inspection and validation in the whole data science workflow. Moreover, as the readability of R is rising rapidly, there seems to be a trend for researchers to communicate and collaborate using R. A well-designed syntax of R language could be comprehended by even non-programmers. In the meantime, these codes could also be run effectively and efficiently in the computer to reproduce the exact same results based on the open shared data. Not only does R provides researchers with powerful computational tools to lower the barrier of statistical implementation, but also provides a big chance to facilitate open science with its wonderful design and community culture [11]. This trend could also create a breeding ground for knowledge transfer and cross-discipline research, as the operational tools and statistical logics underneath could be shared and passed from field to field.

With the joint effort from statisticians, computer scientists and practitioners from versatile backgrounds, the ecosystem of R has become unprecedentedly energetic and diverse in the recent two decades. It is important to acknowledge that the team at RStudio (now Posit) is dedicated to the ongoing development of RStudio and numerous R packages (including the ‘tidyverse’ suite of packages), which have played a significant role in making R more accessible to users all over the world. Through continuous updates and improvements, these tools have streamlined workflows and enhanced the usability of R, particularly for those new to programming and data analysis. While many packages on CRAN are authored by individual developers, this is due to the open-source nature of the community, which allows developers to freely reuse code as long as they adhere to the terms of the license. Therefore, when they start to develop with R and import functions from other packages, they are standing on the shoulders of each other already. This should be considered an advanced form of communication and collaboration. Nevertheless, our investigation shows that there are positive correlations between package author number and the imports, updates and downloads, which indicates that team work might have improved the diversified creativity, development efficiency and software impact in the R development. Lots of local and international communities of R (rOpenSci, RLadies, RStudio, RUGS, etc.), online or offline, commercial or non-commercial, are emerging and running vigorously these years. While they have various scopes and organizational forms, the core of R remains in every one of them, which is to be free, open and collaborative for a better world. Therefore, rather than focusing on league tables such as the TIOBE Index or on direct competition with other programming languages, it is more meaningful to view R as a continuously evolving ecosystem. Rooted in openness and collaboration, R has established a stable and enduring position in data analysis and scientific research, with substantial potential for further growth and innovation.
